# Afferent loop syndrome secondary to recurrent pancreatic adenocarcinoma post-Whipple procedure: case report

**DOI:** 10.3389/fonc.2026.1838218

**Published:** 2026-06-15

**Authors:** Justin Baik, Neil Hughes, Vladimir Ferrer

**Affiliations:** 1 Western Michigan University Homer Stryker MD School of Medicine, Kalamazoo, MI, United States; 2 Gastroenterology, Bronson Methodist Hospital, Kalamazoo, MI, United States

**Keywords:** afferent loop syndrome, endoscopic metal stent, malignant obstruction, pancreatic ductal adenocarcinoma, Whipple procedure

## Abstract

**Background:**

Afferent loop syndrome (ALS) is a rare but serious complication following pancreaticoduodenectomy, most often due to recurrent malignancy.

**Case presentation:**

A 62-year-old male patient with stage IIb pancreatic ductal adenocarcinoma status post-Whipple procedure presented with acute abdominal pain, bowel obstruction, and sepsis after months of persistent watery diarrhea. Imaging demonstrated recurrent pancreatic malignancy causing obstruction of the pancreaticobiliary limb consistent with ALS. Blood cultures grew *Klebsiella pneumoniae*, confirming sepsis secondary to obstruction. Endoscopy revealed a severe non-traversable afferent limb stricture, and palliative decompression with uncovered metal stent placement resulted in rapid clinical improvement.

**Discussion/conclusion:**

This case highlights the need for a high degree of suspicion for ALS in post-Whipple patients with nonspecific gastrointestinal symptoms, particularly in the setting of recurrent malignancy. The prolonged refractory diarrhea preceding acute obstruction illustrates an atypical presentation that may delay diagnosis. Early imaging and minimally invasive endoscopic intervention can provide effective palliation and prevent severe complications.

## Highlights

- Maintain a high index of suspicion for afferent loop syndrome (ALS) in post-Whipple patients with nonspecific obstructive or infectious symptoms.
- Endoscopic decompression with self-expanding metal stents offers an effective, minimally invasive palliative strategy for malignant ALS.
- Recurrent malignancy remains the predominant cause of ALS after pancreaticoduodenectomy, underscoring the importance of multidisciplinary care.

## Introduction/background

Pancreatic ductal adenocarcinoma (PDAC) remains one of the most lethal gastrointestinal malignancies, with a 5-year survival rate of less than 10% despite advances in systemic and surgical therapies (1). Pancreaticoduodenectomy (Whipple procedure) followed by adjuvant chemotherapy remains the only potentially curative approach but carries substantial postoperative morbidity (2). Complications include delayed gastric emptying, pancreatic exocrine insufficiency, nutritional malabsorption, and wound infection (3). Afferent loop syndrome (ALS) is a rare but clinically significant late complication resulting from mechanical obstruction of the afferent limb, which impairs the drainage of bile and pancreatic secretions and manifests as abdominal pain, cholangitis, malabsorption, or bowel obstruction (4). This case report describes a case of post-Whipple ALS secondary to recurrent pancreatic adenocarcinoma. It emphasizes the need for a high degree of suspicion for afferent loop syndrome in post-Whipple patients presenting with nonspecific clinical and laboratory findings. Early recognition is critical to enable timely intervention and prevent progression to severe complications such as sepsis.

## Case description

A 62-year-old male patient with a history of pancreatic ductal adenocarcinoma (pT2N1M0 stage IIb adenocarcinoma) status post-Whipple procedure presented with acute cessation of bowel function and acute onset of abdominal pain. He subsequently had no flatus or stool output and had been unable to tolerate oral intake. Stopping his loperamide nor attempts at bowel preparation with polyethylene glycol, bisacodyl use, or electrolyte drinks did not yield bowel movements, which prompted him to suspect a bowel obstruction and come to the emergency department.

His prior postoperative course was complicated by persistent watery diarrhea despite the use of multiple antidiarrheal agents, which was attributed to dumping syndrome and pancreatic exocrine insufficiency. His stools were entirely watery. Pancreatic enzyme replacement with pancrelipase was not beneficial.

In the emergency department, the patient was hemodynamically stable, but febrile at 102.2°F. The physical examination was remarkable for an ill appearance, a well-healed surgical abdominal incision, absent bowel sounds, and significant tenderness to palpation in the periumbilical and left lower quadrant regions of the abdomen.

Laboratory studies revealed hyponatremia at 132 mmol/L, elevated alkaline phosphatase of 703 U/L, mild transaminase elevations of AST 45 U/L and ALT 58 U/L, anemia at 11 g/dL, and normal white cell count at 5.2 × 10^3^/uL and lactate 1.0 mmol/L. Total bilirubin remained low at 0.8.

CT imaging demonstrated new ill-defined soft tissue surrounding the superior mesenteric artery and vein, hepatic lesions suggestive of metastases, and distention of the pancreaticobiliary limb consistent with obstruction (**Figure 1**). Blood cultures grew *Klebsiella pneumoniae*, confirming sepsis from an intra-abdominal source. MRI/MRCP further delineated the recurrent neoplasm encasing the superior mesenteric artery with secondary biliary and pancreatic limb obstruction (**Figure 2**). Endoscopic evaluation confirmed a severe non-traversable extrinsic stricture of the afferent limb. For palliative management, a 22 mm × 12 cm uncovered metal stent was placed using a guidewire across the structuring defect, resulting in copious amounts of bile flowing through the stent. This led to immediate decompression and resolution of sepsis.

**Figure 1:**
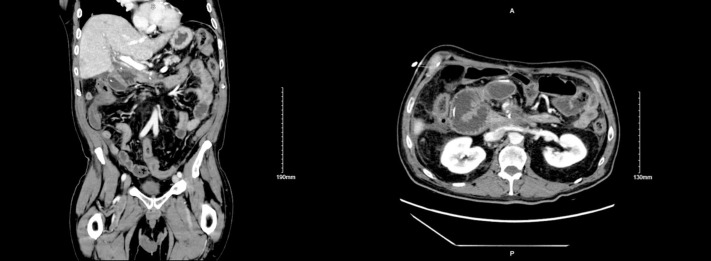
CT of abdomen and pelvis with contrast showing ill-defined soft tissue along the superior mesenteric vessels and distention of the pancreatico-jejunal enteric arm.

**Figure 2:**
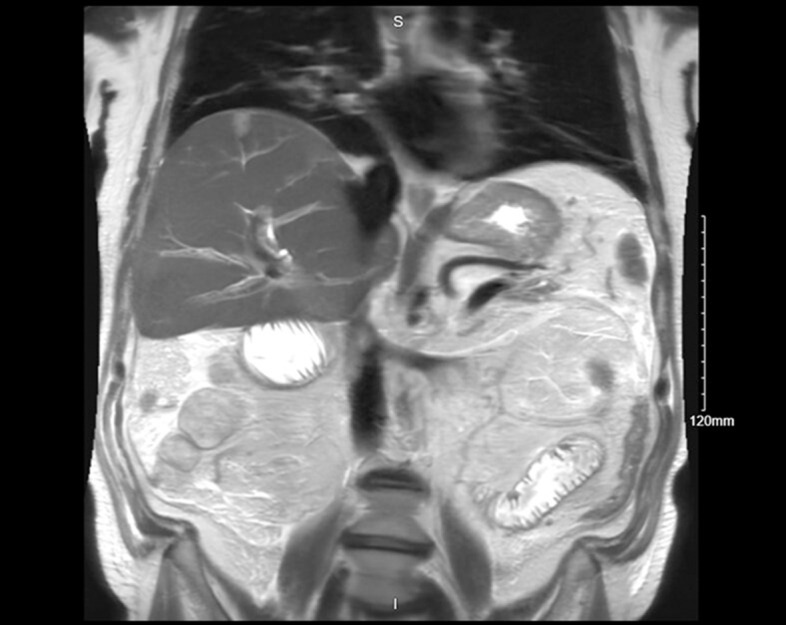
MRI/MRCP showing hypoattenuation within the liver along the right hepatic dome consistent with metastatic disease.

The patient was treated with broad-spectrum antibiotics targeting *Klebsiella* bacteremia, electrolyte repletion, nutritional optimization, and pain management. Endoscopic decompression with self-expanding metal stent placement provided rapid clinical improvement. Given the presence of metastatic disease and limited surgical options, management was focused on palliation. Following a multidisciplinary discussion, the patient’s care plan was transitioned to comfort-focused therapy, with palliative and advanced illness management services engaged. Hence, no further endoscopic procedures or images were pursued. The patient had persistent diarrhea and elevated liver function tests, which were monitored in the outpatient setting, and oral antibiotics were continued upon discharge for 2 weeks after the first negative culture result.

## Discussion

Afferent loop syndrome (ALS) occurs in 0.2%–1% of patients following pancreaticoduodenectomy, most commonly due to recurrent malignancy (5). To the best of the author’s knowledge, only a few cases of confirmed ALS cases from pancreatic cancer recurrence in post-Whipple patients have been reported in the literature (5–8). Obstruction of the afferent limb impedes the flow of bile and pancreatic secretions, leading to progressive distension, cholangitis, and risk of intestinal ischemia or perforation (8).

The patient’s longstanding diarrhea prior to presentation was multifactorial. Post-Whipple diarrhea is often attributable to rapid nutrient transit (dumping syndrome), pancreatic exocrine insufficiency, or small intestinal bacterial overgrowth. In ALS, functional exclusion of pancreatic enzymes from the alimentary tract exacerbates malabsorption and steatorrhea (9). The multifactorial nature of post-Whipple diarrhea explains the persistence of watery stools despite enzyme supplementation with pancrelipase.

The diagnosis of afferent loop syndrome relies on a combination of high clinical suspicion and cross-sectional imaging, with CT or MRCP demonstrating the dilated afferent limb and obstructing lesion. Endoscopy provides both confirmation and an avenue for intervention, particularly when strictures are visualized but non-traversable (4).

Definitive treatment of ALS depends on etiology. Surgical revision offers durable outcomes but is often impractical in patients with end-stage malignancy, significant comorbidities, or advanced age. Since recurrent cancers are considered advanced stage, palliative treatment with endoscopic intervention remains the primary management approach (4). In this case, endoscopic decompression resulted in rapid clinical improvement and enhanced quality of life, consistent with prior literature describing a median survival of approximately 5 months post-stent placement (10). More recent case reports have favored endoscopic metal stent placement (6) over surgical decompression (5, 7, 8), suggesting a shift toward less invasive palliative management strategies in patients with advanced malignancy and significant operative risk. Endoscopic ultrasound-guided gastroenterostomy with lumen-apposing metal stent placement has also emerged as a promising minimally invasive option for malignant ALS, with recent studies demonstrating high technical and clinical success rates, improved quality of life, and lower reintervention rates compared with percutaneous drainage or enteral stenting (11–13).

In conclusion, this case underscores the importance of recognizing afferent loop syndrome as a rare but serious complication of Whipple surgery, particularly when driven by the recurrence of malignancy. It highlights the multifactorial nature of post-Whipple diarrhea, the central role of imaging in diagnosis, and the value of endoscopic stenting as an effective palliative method in patients with advanced disease. Early recognition and multidisciplinary management are essential to improving symptom control and quality of life.

## Data Availability

The original contributions presented in the study are included in the article/supplementary material. Further inquiries can be directed to the corresponding author.
